# Isaria tenuipes Peck, an entomopathogenic fungus from Darjeeling Himalaya: Evaluation of in-vitro antiproliferative and antioxidant potential of its mycelium extract

**DOI:** 10.1186/s12906-020-02973-w

**Published:** 2020-06-11

**Authors:** Dhani Raj Chhetri, Abhijit Chhetri, Nerina Shahi, Snigdha Tiwari, Shibendra Kumar Lal Karna, Dorjay Lama, Yuba Raj Pokharel

**Affiliations:** 1 Biochemistry and Molecular Biology Lab, Department of Botany, Sikkim University, Gangtok, Sikkim 737102 India; 2 Cancer Biology Laboratory, Faculty of Life Science and Biotechnology, South Asian University, Chanakyapuri, New Delhi, 110021 India; 3 National Fungal Culture Collection of India, Biodiversity and Palaeobiology Group, MACS’ Agharkar Research Institute, G.G. Agarkar Road, Pune, 411004 India; 4 Department of Microbiology, St. Joseph’s College, North Point, Darjeeling, West Bengal 734104 India; 5 Centre for Health and Disease Studies Nepal, P.O. Box No. 9503, Sankhmul, Baneshwor, Kathmandu, Nepal

**Keywords:** Antioxidant, antiproliferative activities, Cytotoxicity, FTIR, *Isaria tenuipes*

## Abstract

**Background:**

*Isaria tenuipes* is one of the potent species in the members of the genus *Isaria*, which is well reported to possess multiple bioactive substances of therapeutic importance. Therefore*,* an in vitro experimental study was carried to evaluate the bioactivities of the crude methanolic extract from the mycelium of this fungus.

**Methods:**

The fungus was authenticated through morphological characters and the species discrepancy was resolved using the nuclear rDNA ITS sequence. The methanolic extract was fingerprinted by FTIR. The antioxidant components in terms of total phenols and flavonoids were determined as gallic acid and quercetin equivalents respectively. Antioxidant activities of the methanolic extract was assessed using 1, 1-diphenyl-2-picrylhydrazyl (DPPH), 2, 2^/^-azinobis-(3-ethylbenzthiazoline-6-sulphonic acid) radical cation (ABTS^0+^), Fe^2+^chelating activity, and hydroxyl radical scavenging assays. Cytotoxicity of the extract was determined by [3-(4, 5-dimethylthiazol-2-yl)-2, 5-diphenyltetrazolium bromide] (MTT) assay on three cancer cell lines: HeLa, HepG2, and PC3. Apoptosis was further studied by propidium iodide (PI) and Annexin-V/PI staining flow cytometric analysis. Anti-proliferation capacity was studied by colony-forming assay.

**Results:**

In the present study total phenol content of the dried methanol extract was 148.09 ± 3.51μg gallic acid equivalent/mg and flavonoid was 9.02±0.95 μg quercetin/mg. The antioxidant activities of methanol-water extract (8:2 v/v) from cultured mycelia of *I. tenuipes* investigated and evaluated with 1, 1-diphenyl-2-picrylhydrazyl (DPPH) radical scavenging assay revealed IC_50_ value of 5.04mg/ml with an inhibition rate of 74.77% at 10mg/ml and with an iron-chelating assay the chelating ability was recorded to be 86.76% where the IC_50_ value was 4.43 mg/ml. In comparison among the antioxidant assays, 2,2^/^-azinobis-(3-ethylbenzthiazoline-6-sulphonic acid) radical cation (ABTS^0+^) and hydroxyl assay exhibited radical scavenging rate of 44.42% and 49.82% respectively at a concentration of 10 mg/ml. The IC_50_ value of the extract in MTT assay was 43.45μg/ml with HeLa cells, 119.33μg/ml with PC3 cells, and 125.55μg/ml with HepG2 cells.

**Conclusion:**

In this study, it can be concluded that the crude methanolic extract exhibited potent antioxidant and antiproliferative activities suggesting natural antioxidative and antiproliferative agents.

## Background

Darjeeling Himalaya is situated between the 87°59′– 88°53′E and 28°31′–27°13′N in the Eastern Himalayan region of India. This region is characterized by a wide array of climatic zones which favour a luxuriant growth of vegetation of diverse life forms, habits, genus, species, varieties and ecotypes. This region is also the abode of many endemic elements [1]. Due to the presence of its exceptional ecological variations, it is also one of the important regions of fungal resources with overwhelming economic and gastronomic relevance [2]. This region remains unexplored regarding the studies on the genus *Isaria*. Therefore, the present study was conducted to determine the in vitro antioxidant and anticancer activities of *Isaria tenuipes* from its methanolic mycelial extract.

Entomopathogenic fungi are a kind of fungal pathogens that infect different types of insects (arthropods). Some entomopathogenic fungi have been used to develop mycopesticides for agricultural use [3]. On the other hand, some other species produce multiple secondary metabolites with bioactivities that have the potential for medicines or nutriments [4]. Therefore, of late, the search for bioactive compounds from insect pathogenic fungi has shown increased interest [5]. *Isaria tenuipes* Peck (synonym: *Paecilomyces tenuipes* Peck Samson) is a multi-infectious fungus parasitizing insects of the order Lepidoptera [6]. The fungus has been considered as a producer of diverse bioactive compounds such as isariotins, beauvericin, beauveriolides, and fingolimod [7–9].

Several entomopathogenic fungi such as *Cordyceps* species are popular as medicinal mushrooms and have been used for traditional health foods and medicines for a long time in Asia [10]. Chinese herbal medicine system believed that *Isaria sinclairii* may herald eternal youth [11]. *Isaria tenuipes* has been used as folk medicine or health food in Japan, China, and South Korea [12]. It is being used for hundreds of years as a food ingredient to strengthen the immune system and regain energy [13]. In the Eastern Himalayan region, *Isaria tenuipes* is traditionally used as a tonic and food supplement for recovery from tuberculosis and for speedy recuperation after childbirth.

Oxidative stress is a two-sided process. On one hand, excessive oxidant challenge results in damage to biomolecules. On the other hand maintenance of a physiological level of oxidant challenge, termed oxidative eustress, is very essential for leading life processes through redox signaling [14]. Therefore, oxidative stress generated due to the imbalance between reactive oxygen species (ROS) and antioxidative protection, going in favor of the former, is responsible for most of the major diseases [15]. Cancer is one of the leading causes of death worldwide and reports suggest that cancer cells are under continuous oxidative stress [16] due to the generation of ROS. Research carried out with human tumor cell lines indicates that cancer cells produce ROS at a much higher rate than healthy cells [17]. Antioxidants play an important role by inhibiting the oxidation of biomolecules as well as scavenging various free radicals. Therefore, natural antioxidants are popular for its therapeutic efficacy and have been extensively studied which makes prospecting for bioactive mushroom products an important area of research [18]. Besides its use in various diseases elicited by oxidative stress, many of the species of mushrooms have received considerable impetus for their biological activities such as antitumor, anti-inflammatory, and immunological activities [19].

Medicinal mushrooms have various biological activities and can suppress the proliferation of many types of cancer cells such as breast cancer, hepatocellular carcinoma, uterine cervix cancer, pancreatic cancer, gastric cancer, and acute leukemia cells [18]. In recent years entomopathogenic fungal metabolites have attracted attention since they exhibit a wide variety of insecticidal, antimicrobial, anticancer, antioxidant, and antiviral activities. Furthermore, they have been suggested as potential candidates for the development of new bioactive agents [20]. It is also well established that many species of entomopathogenic fungus such as *Isaria farinosa* possesses anti-oxidative properties as well as the capacity for inhibiting cancer growth [21]. In this backdrop, special attention has been paid to assess the antioxidant and antiproliferative activities of the mycelial extract of an entomopathogenic fungus *Isaria tenuipes*.

## Methods

### Identification of fungus using micro-morphological characters and DNA sequencing analysis

**Fig. 1:**
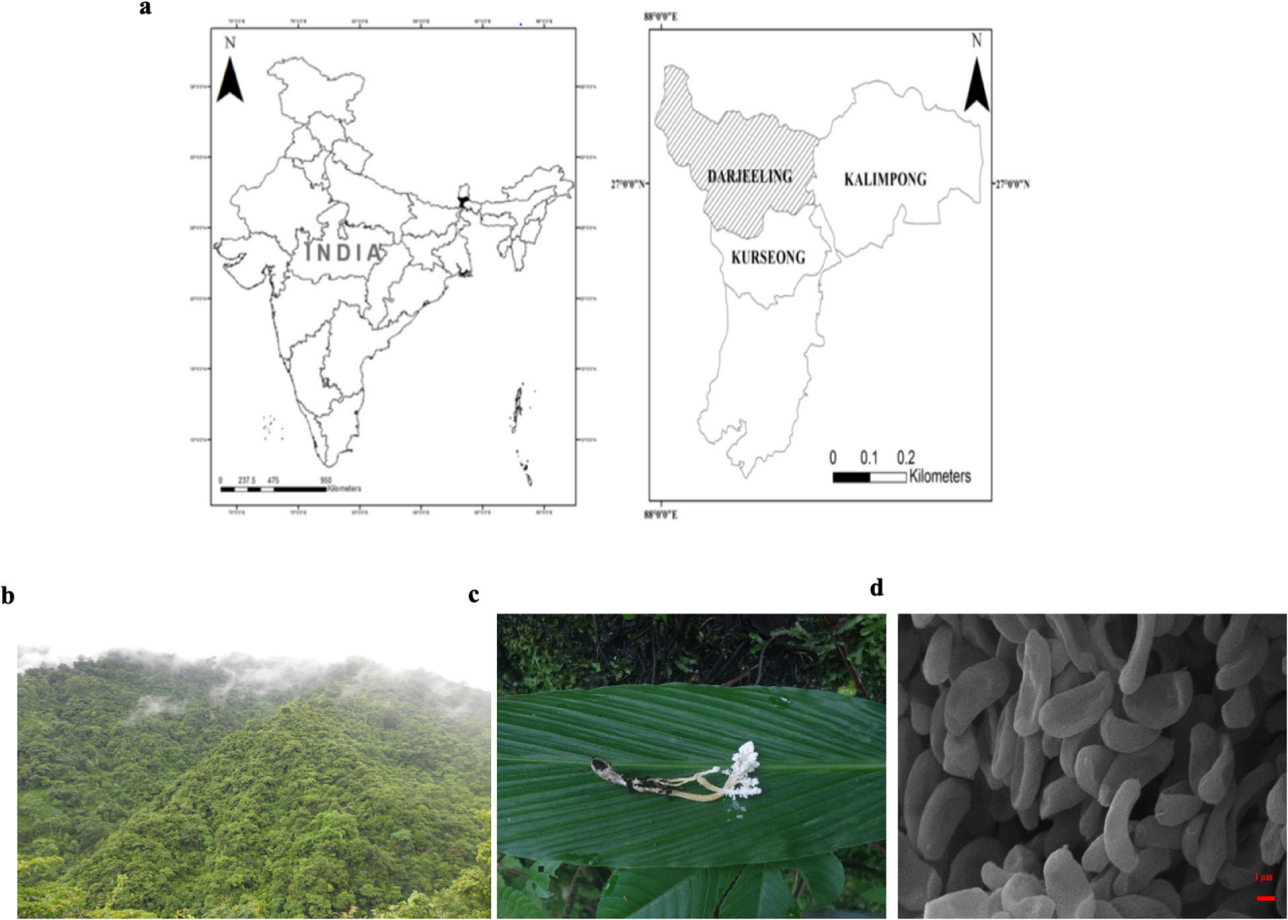
**a-d** Habitat and habit of *Isaria tenuipes***: a**. Map of Darjeeling Himalaya; **b**. Habitat of *Isaria tenuipes* in Darjeeling Himalaya; **c**. Wild fruit bodies of *Isaria tenuipes*; **d** SEM image of conidia**.** Map developed using Arc. GIS 10.1 shape file downloaded from world clim database (www.world climate. org)

Sample of *Isaria tenuipes* Peck (Fig. 1) was collected from Lebong, a region in Darjeeling region during September 2018. After collection, the fungus was aseptically brought to the laboratory and single spore isolation was made on agar media. Taxonomic identification of the collected fungus was made according to taxonomic keys and monographs following the standard protocol described by Luangsa-sard *et al.,* [22].

For molecular identification, genomic DNA was extracted from pure axenic cultures grown on potato dextrose agar (PDA) for one week at 25°C, by a simple and rapid DNA extraction protocol [23] using FasPrep 24 tissue homogenizer (MP Biomedicals GmbH, Eschwege, Germany). The DNA was resuspended in 50 μL TE buffer and analyzed quantitatively as well as qualitatively by 1 % agarose gel electrophoresis. The nuclear ribosomal internal transcribed spacer (ITS) gene was amplified by PCR using primer pair ITS4 & ITS5 [24] in a reaction volume of 50 μL. The reaction mixture contained 32 μL PCR grade water (Sigma, St. Louis, MO, USA), 5μL PCR buffer (10×), 4μL of 10 mMdNTPs mix (Sigma-Aldrich), 1 μL of each primer (20 pmol/μL), 1 μL (5 U/μL) of Taq polymerase (Sigma-Aldrich) along with 20–50 ng of template DNA. Amplification was done using an Applied BiosystemsProFlex PCR System (Applied Biosystems, Waltham, MA, USA) following standard cycling conditions: initial denaturation at 95°C for 5 min, followed by 35 cycles of denaturation at 95°C for 90 seconds, primer annealing at 52°C, primer extension at 72°C for 1 min, and a final extension step at 72 °C for 10 min. The amplified products were analyzed on 1.2% agarose gel containing ethidium bromide. The PCR products were purified using an Axygen PCR cleanup kit (Axygen Scientific, CA, USA). Sequencing reactions were performed with a BigDye terminator cycle sequencing kit, ver. 3.1/1.1 (Applied Biosystems). All the sequencing reactions were purified and analyzed on an ABI Avant 3100 automated DNA sequencer (Applied Biosystems).

### Preparation of the fungal extract

Fungal mycelium was grown by static liquid culture in 2l Erlenmeyer flasks, the mycelium biomass was separated by filtration with Whatman No 1 filter paper and washed thoroughly with deionized H_2_O. The mycelium biomass was dried at 50^o^C for 36 hours, powdered, sieved through 0.1mm mesh, and stored at 4°C until further use. 5 gram powdered mycelium was extracted with 10 vol. of 80% methanol at room temperature for 6 hours with continuous stirring. The extraction was repeated thrice using fresh solvents, and the combined extracts were pooled together and the extract was evaporated to dryness at low temperature in a rotary evaporator. The dried extract thus obtained was re-dissolved in methanol (ITM) at a concentration of 10mg/ml and then diluted to 2, 4, 6, and 8 mg/ml for antioxidant analyses. The yield of the extract was 15± 0.25% on a dry weight basis.

### Determination of total phenols and flavonoids content

The total phenol content of the fungal extracts was assessed using the Folin–Ciocalteu reagent method described by Singleton and Rossi [25] with minor modification. Briefly, the fungal extract (0.5ml) was mixed with 0.5 ml of 10% (v/v) Folin–Ciocalteu reagent and dark incubated for 5 min at room temperature (RT). After that, 1 ml of freshly prepared sodium carbonate (10% w/v aqueous solution) was added and mixed well. After 10 min of dark incubation, the absorbance of the blue color that developed was read at 730 nm. Gallic acid (10-100 μg/ml) was used as the phenolic standard and the total phenolic content was expressed as gallic acid equivalents (GAE). All tests were carried out in triplicates.

The total flavonoid content of all the fungal extract was quantified according to the colorimetric method described by Zhishen *et al*. [26] with minor modification. About 250μl of the fungal extract was mixed with 1.25 ml deionized water and 75 μl of 5% NaNO_2_. After 5 min of incubation at room temperature, 0.15 ml of 10% AlCl_3_ was added and mixed well. The mixture was incubated for 6 min at room temperature and 0.5 ml of 1 mM NaOH. Finally, the volume was made up to 2.5ml with 275 μl of deionized water and mixed well. The absorbance was measured at 510 nm after 30 minutes of incubation. Quantification of flavonoids was done from a quercetin standard curve and results were expressed as quercetin equivalents (QtE). All tests were carried out in triplicates.

### DPPH (2, 2-diphenyl-1-picrylhydrazyl) radical scavenging assay

The free radical scavenging activity of the methanol extracts on the stable radical 2,2-diphenyl-1-picrylhydrazyl (DPPH) was evaluated by the method of Brand- Williams *et al.,*[27] with some modifications. Briefly, increasing concentrations of fungal extracts (1 ml) were mixed with 3 ml of the methanolic solution of DPPH and adjusted to the final absorbance of DPPH to 1.0 (±0.02) at 517nm.The reaction mixture was shaken thoroughly and then kept in the dark at room temperature for 30 min and absorbance was measured at 517 nm against a blank. The percentage of the DPPH radical scavenging was calculated with the following equation:

*Scavenging activity* (%) = [*Ac* − *As*)/*Ac*] 100

Where *Ac* = absorbance of the control (8:2 v/v MeOH-H_2_O in place of the sample) and *As* = absorbance of the sample.

The IC_50_ value represented the concentration of the methanolic extract that caused 50% inhibition of DPPH radical formation was determined by interpolation from a linear regression analysis of methanol extract (2 –10 mg mL^-1^).

### ABTS Radical Scavenging Activity

The scavenging activity of cationic ABTS radical was determined using the method of Re *et al*., [28]. Briefly, 7 mM of ABTS stock solution was mixed with 2.45mM potassium persulfate solutions prepared in deionized water. The reaction mixture after sixteen hours of reaction in dark generates ABTS radical cation. The ABTS ^● +^ solution was suitably diluted with ethanol to yield an absorbance of 0.70 (± 0.2) at 734nm and equilibrated at 25°C to be used for the antioxidant assay. The assay was performed by adding 1ml methanolic extracts to be tested at different amounts to 3ml of ABTS^●+^ radical cation solution and the mixture was shaken gently, incubated for six minutes at 37°C. The reduction of ABTS^●+^ radical cation absorbance by adding compounds that contain antioxidants was measured by the change of absorbance of ABTS^●+^ radical cation at 734nm using methanol as a blank, on UV- visible spectrophotometer (Thermo Fisher Scientific). A standard solution of ascorbic acid was also prepared and tested at a range of 2 to 10mg/ml in methanol (HPLC grade, HiMedia). The percentage inhibition was calculated using the formula:

$$ Scavenging\ activity\ \left(\%\right)=\left[ Ac- As\right)/ Ac\Big]\ 100 $$

Where, *Ac* and *As* is the absorbance of control and sample, respectively. The result was compared with control which was prepared by adding 1.0ml of methanol in place of the sample.

### Evaluation of metal-chelating activity

The chelating effect on ferrous ions by the extracts was determined according to the method of Jiang *et al.,* [29] with slight modifications. To a 1-ml aliquot of a methanol extract (2–10 mg/ml) was mixed with 3.7ml deionized water. To the reaction mixture, 0.1ml of 2.0 mM aqueous ferrous chloride (Merck) and 0.2ml of 5.0 mM ferrozine (Sigma) was added. After 10 minutes, absorbance at 562 nm was determined spectrophotometrically. Ethylenediaminetetraacetic acid (EDTA) was used as a positive control. A lower absorbance indicated a higher Fe^2+^-chelating ability. The percentage chelating capacity was calculated as;

*Chelating activity* (%) = [*Ac* − *As*)/*Ac*] 100

Where *Ac* was the absorbance of the control in the reaction system and *As* was the absorbance of the sample.

### Hydroxyl radical scavenging assay

The hydroxyl radicals for the assay were generated in an H_2_O_2_ –FeSO_4_ system by oxidation of FeSO_4_ and were assayed by the color change of salicylic acid according to the method of Zhong *et al*., [30] with slight modifications. The hydroxyl radical was produced in a reaction mixture containing 1 ml of sample (2–10 mg/ml), 1 ml of 9 mM FeSO_4_ and 1 ml of 0.3% H_2_O_2_ in 0.5 ml of 9 mM salicylic acid–ethanol solutions were mixed well and the mixture was incubated at 37°C for 30 min. The change in absorbance caused by the color change of salicylic acid was recorded at 510 nm. Gallic Acid was used as positive control. The hydroxyl radical scavenging activity was calculated as follows:

$$ Scavenging\ activity\ \left(\%\right)=\left[ Ac- As\right)/ Ac\Big]\ 100 $$

Where *Ac* is the absorbance of the control (methanol instead of the sample), *As* is the absorbance of the sample.

### Cell viability assay

Three human carcinoma cell lines viz., HeLa (cervical cancer), PC3 (prostate cancer), and HepG2 (hepatocarcinoma) were used to investigate the cytotoxic activity evaluation of the methanolic fungal extract. The cells were cultured to reach confluence in DMEM and RPMI1640 supplemented with 10% FBS, and 100 unit/ml penicillin, and 100 μg/ml streptomycin and maintained at 37 °C in a humidified atmosphere with 5% CO_2_ incubator.

The influence of the methanolic extract on cell viability was assessed by the MTT assay as described by Mossman [31]. MTT [3-(4,5-dimethylthiazol-2-yl)-2,5-diphenyltetrazolium bromide] is a tetrazolium salt that appears yellow in color in its oxidized form which when cleaved by mitochondrial and endoplasmic reticulum dehydrogenase yields a measurable purple formazan product. This formazan production can be quantified spectrophotometrically and is proportional to the viable cell number or inversely proportional to the degree of cytotoxicity [32].

Exponentially growing cultured cells were trypsinized, counted, and seeded at a density of 3 × 10^3^ cells/well in a 96-well plate. After 24 hr adherence, the cells were treated with various concentrations of fungal extract for 72 hr. After, the incubation, 20μl of MTT solution (5 mg/mL) was added to each well and incubated for 3 hr in the dark. Then the medium was carefully removed, and the formazan formed in the wells was dissolved in 150 μl of dimethyl sulfoxide and the plates were kept for 5 min on a plate shaker. The absorbance was measured at 570 nm using a microplate reader. All the experiments were performed in four replicates.

### Determination of cell apoptosis by PI staining

The HeLa cells in the logarithmic growth phase were suspended at a final concentration of 3x10^4^/mL in a 12-well culture plate. After the desired period of incubation, the cells were treated with different concentrations of methanolic extracts for 72 hours. Then, an aliquot of the treated cell was harvested, trypsinized, centrifuged at 4^o^C, 1500 rpm, supernatant aspirated off and the pellet washed with PBS. Pellet was further resuspended on ice-cold PBS in which 5μl of PI solution was added briefly mixed and incubated on ice under dark conditions for 20 minutes. Then the cell apoptosis was analyzed by using a flow cytometer (BD FACS Verse) and the gate was used to exclude any clumped nuclei. The PI staining was carried out only in HeLa cell lines as this cell exhibited IC_50_(>50) value within the range of extract concentration tested.

### Annexin-V/PI Staining

Apoptosis was measured by flow cytometry using Annexin-V/PI double staining. Hela cells were seeded in a 6-well plate at a density of 0.50 *10^6^ cells/well and incubated at 37°C for 24 h. Then, the medium was removed and fresh media with the indicated concentrations of extracts were added. After 48h, cells were collected and washed with ice-cold PBS twice and resuspended in 25μl of 1X Annexin-V binding buffer, 1.5μl of Annexin-V staining solution 10μl of PI (stock: 50 μl/ml),(BD Biosciences) staining solution and incubated on ice under the dark condition for 20 min in dark. Then, the number of viable, apoptotic, and necrotic cells were quantified using flow cytometry.

### Colony Forming Assay

To check the long term effect of the extracts on Hela cells, cells were seeded in a 6 well plate at a density of 3000cells/well and incubated at 37°C for 24h. Then, the media was removed and fresh media with the indicated concentrations of extracts were added. Every other two days, media was removed and fresh media with desired concentrations of extracts were added for at least 10 days. After ten days, media was removed, each well was washed with PBS, fixed with methanol for 20 min followed by washing with PBS and staining with 0.4% crystal violet for 30 min. After staining, each well were rinsed with tap water to remove the excess stain and the image was analyzed using Image J software.

### HPLC analysis

Analysis of beauvericin in the extract was done in an HPLC system (Dionex, Ultimate 300) using a reversed-phase HPLC column (Thermo Scientific, 2.1 x 150 mm) with isocratic conditions and a mobile phase of acetonitrile-water (75:25) at a flow rate of 0.5ml/min with UV detection at 210 nm for fifteen minutes. Precise quantities of beauvericin (Sigma, USA) were dissolved in methanol as an internal standard. Beauvericin was determined by comparing peak areas from sample to an internal standard. All reagents used for HPLC analysis were degassed and sterilized using a 0.22 μm syringe filter.

### Morphological evaluation of apoptosis using light microscopy

The morphological changes of HeLa cells were determined using an inverted light microscope. HeLa cells were grown in 12 well plates and treated with mycelial methanolic extract at a concentration of 100μg/ml for 72 h. After incubation under optimal conditions, the morphological changes were examined at 4X under an inverted light microscope (Nikon TS100).

### FT-IR spectroscopy

The IR spectrum of methanolic extract was analyzed using Fourier transform infrared spectroscopy (Perkin Elmer Spectrum 1: FT-IR), having a resolution of 1.0 cm^-1^. The infrared spectra of extract were recorded using potassium bromide (KBr) pellets covering the scan range from an entire region of around 4000- 450 cm^-1^.

### Statistics

All results are expressed as mean ± standard deviation values of the three sets of observations. MTT and Annexin-V/PI assays were analyzed using one-way ANOVA followed by turkey's post hoc test of significance (where different alphabets denote significant difference (*p*<0.05)).

## RESULTS

### Identification of an entomopathogenic fungus

Entomopathogenic fungus *Isaria tenuipes* was grown in potato dextrose agar. The colony morphology of the fungus was white to cream-colored, with less compact mycelial growth reaching 32±1 mm at 24°C in 15 days of dark incubation. Conidia were ellipsoidal to cylindrical, often slightly curved, and measured 3.75-4.5 x 1.3-2.25 μm (average 4.1 x 1.7 μm) on the host (Fig. 1d). Further, BLAST analysis based on the nuclear rDNA ITS sequence data was carried out to confirm the identity of the fungus. Based on BLAST analysis of the nuclear rDNA ITS sequence, the fungus was successfully identified as *Isaria tenuipes* with 100% similarity.

### Total phenolic (TPC), flavonoid contents (TFC) and beavurecin

The methanolic extract of *Isaria tenuipes* (ITM) revealed the presence of appreciable quantities of total phenolic and total flavonoids as antioxidant components which could be useful to trap free radicals. The phenol content was determined based on the calibration curve of gallic acid (10-100 μg/ml). Similarly, the calibration curve of quercetin (1-100 μg/ml) was used to determine the flavonoid content. Beauvericin was quantified with standard beavurecin using HPLC Table 1.

**Table 1:** Antioxidant constituents present in mycelium extract

Fungus	Mycelium extract	Total phenol μg GAE g^**-1**^	Total flavonoid mg QE g^**-1**^	Beauvericin (μg/ml)
***Isaria tenuipes***	**Aqueous: Methanol (2:8 v/v)**	**148.09 ± 3.511**	**9.02 ±2.09**	**6.71± 0.31**

### Antioxidant Activities of ITM

**Fig. 2:**
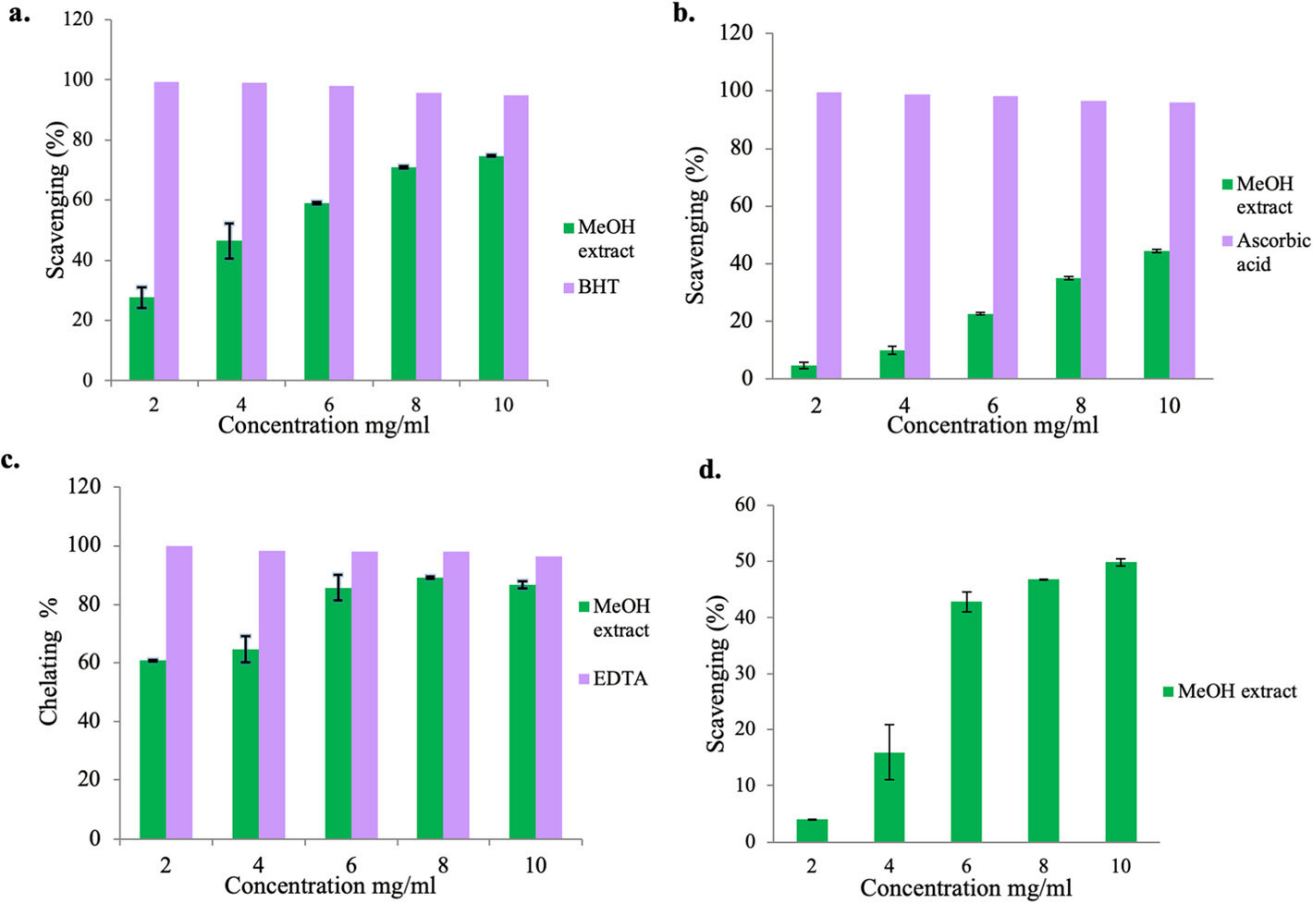
**a-d.** Antioxidant activity of varying concentrations of methanolic extract of *I. tenuipes* assayed through different assay methods: **a**. DPPH radical scavenging activity (BHT was used as standard); **b**. ABTS radical scavenging activity (ascorbic acid was used as standard); **c**. Fe^2+^ chelating potential (EDTA was used as standard) and (**d**). Hydroxyl radical scavenging activity

The scavenging effect of the methanolic extract on the DPPH radical was highly pronounced in the case of reference standard BHT. The scavenging effect of the methanol extract was directly proportional to its concentration (2-10 mg/ml). At this range of concentration 27.56-74.77 % DPPH radicals were scavenged (Fig. 2a). Further, the scavenging activity was recorded to be maximums at the highest concentration tested. IC_50_ value of the extract was determined to be 4.97 mg/ml.

An experiment performed to determine the ABTS^•+^ radical scavenging effect of the methanolic extract revealed significant activity in ascending order (Fig. 2b). The crude methanol extract proved to be an excellent ABTS^•+^ radical decolorization antioxidant in a dose-dependent manner. However, at the tested concentrations, the inhibition was found from 4.77- 44.42 %. However, with similar concentrations, ascorbic acid showed a better scavenging activity.

To assess the chelating properties of the crude methanol extract, the disruption of Fe^2+^-ferrozine complex at various concentrations was estimated and compared with the chelating standard, EDTA. In this assay, the methanolic extract demonstrated the ability to interfere with the formation of the ferrous-ferrozine complex at all the tested concentrations. The result suggests the extract has an appreciable chelating activity which increased with increasing extract concentrations reaching up to 86.76% chelation at 10mg/ml (Fig. 2c).

The hydroxyl radical was determined based on the principle of Fenton reaction and scavenging potential assayed by the oxidation of salicylic acid. Hydroxyl scavenging effect of methanolic extracts increased with concentrations and was low to moderate (4.0 –49.82%) at 2 to 10 mg/ ml (Fig. 2d). Results indicated that methanol extract had the moderate ability of hydroxyl scavenging, which was comparatively lower concerning other assays.

### FTIR spectrum

**Fig. 3:**
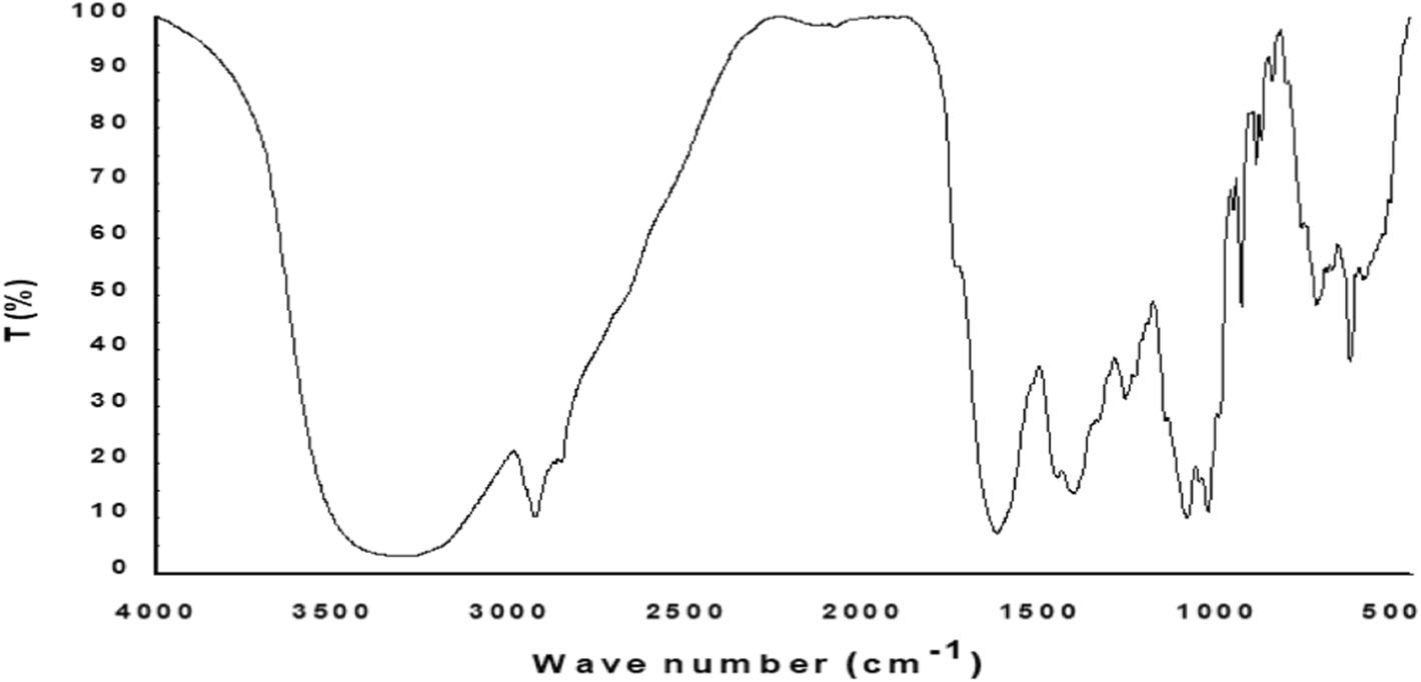
Fourier transform infrared spectra of methanolic extract of *Isaria tenuipes* prepared as KBr pellet and scanned in the range 4000 to 450 cm−1

To examine the probable chemical compositions of the crude methanolic extract, FTIR spectroscopy was used. The FTIR spectrum analysis (Fig. 3) of the lyophilized extract of *Isaria tenuipes* shows the characteristic absorption peaks at 3292 cm^−1^ (γ -N-H stretch), 2931 cm^−1^ (aliphatic C-H stretching, asymmetric) and 2855 cm^−1^ (C-H stretching, symmetric) respectively. The vibration at 1622 cm^−1^ indicates γ C=N stretching and γ N-O (cis) stretching at 1405 cm^-1^ whereas the peak at 931 indicates γ N-O stretch. The results showed the presence of N–H stretching vibration, C-H stretching, C=N stretching and N-O stretching vibration which could indicate the presence of some alkene, amine, and nitro compounds in methanolic extract.

### Anti-proliferative activities of ITM in Hela, HepG2 and PC3 cancer cell lines

**Fig. 4:**
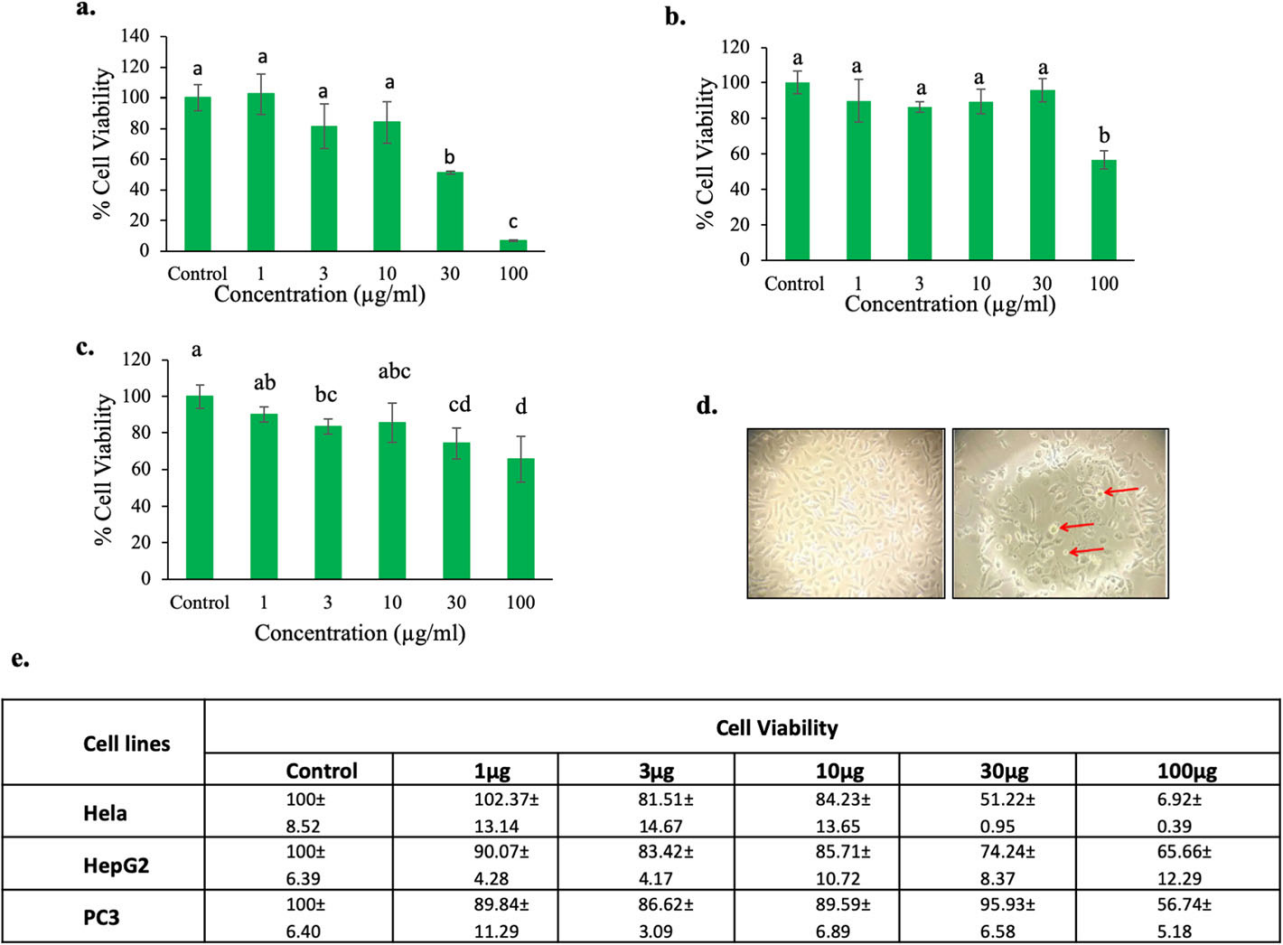
Effect of ITM at different concentrations on cancer cell viability: **a** HeLa, (**b**) PC3, and (**c**) HepG2 cell lines. The picture shows Light micrograph image of untreated HeLa cells (left) Methanol extract (100μg/ml) (right) treated HeLa cells after 72 hours, arrow showing apoptotic bodies (**d**). The table shows the mean standard deviation (SD) percentage of viable cells from three independent experiments (**e**). Data are expressed as a mean ± SD (n=3). ^a~d^ values with different letters were significantly different at *p* < 0.05, as analyzed by one-way ANOVA followed by Turkeys post hoc test of significance

Cytotoxic activity of ITM was determined by the MTT assay against three human carcinoma cells. These three cell lines, HeLa, HepG2, and PC3 were exposed to increasing concentration of the extract. The percentage inhibition of the three different cancer cells when cultured along with the varied concentration of methanolic extract is shown in Fig. 4 a-e. The ITM had the most effective dose-related cytotoxic activities on HeLa cell proliferation with 93.41% inhibition of exponentially growing cultured cells at a concentration of 100μg/ml with IC_50_ value of 43.45μg/ml, within 72 hours of treatment as shown in Fig. 4a. The inhibition of exponentially growing cultured HepG2 and PC3 cells showed 39.42% and 26.01% inhibition at a tested concentration of 100 μg/ml respectively as shown in Fig. 4b and c. The standard deviation of cell viability assay was calculated for all three cell lines as shown in Fig. 4e.

**Fig. 5:**
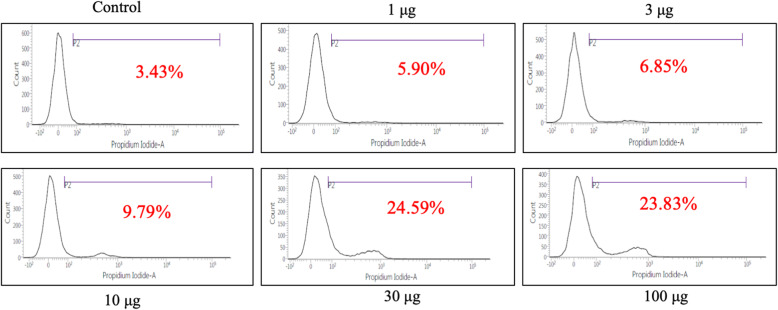
Cell death was assessed by flow cytometry of PI staining. Cells were incubated with untreated and 1-100 μg/ml methanol extracts for 72 h. Exposure to 30 and 100μg/ml methanol extract results in an increased level of cell death

Determination of cell apoptosis by the propidium iodide (PI) flow cytometric assay is based on the principle that apoptotic cells, among other typical features, are characterized by DNA fragmentation and, consequently, loss of nuclear DNA content. Flow cytometric method may determine the cause of cell death and since the methanolic extract exhibited promising results with HeLa cell line in the MTT assay, we further conducted the propidium iodide (PI) staining to evaluate apoptosis in the HeLa cells. In the present study, the result of PI staining demonstrated that the percentage of apoptosis in HeLa cells treated with 1, 3, 10, 30 and 100 μg/ml concentrations of methanolic extract was 5.90% 6.85%, 10.67% 24.59% and 23.83% respectively (Fig. 5). Besides, the control sample without treatment exhibited that 0.54% of cells were undergoing apoptosis in contrast to the cells treated with DMSO, where it was 3.43%.

**Fig. 6:**
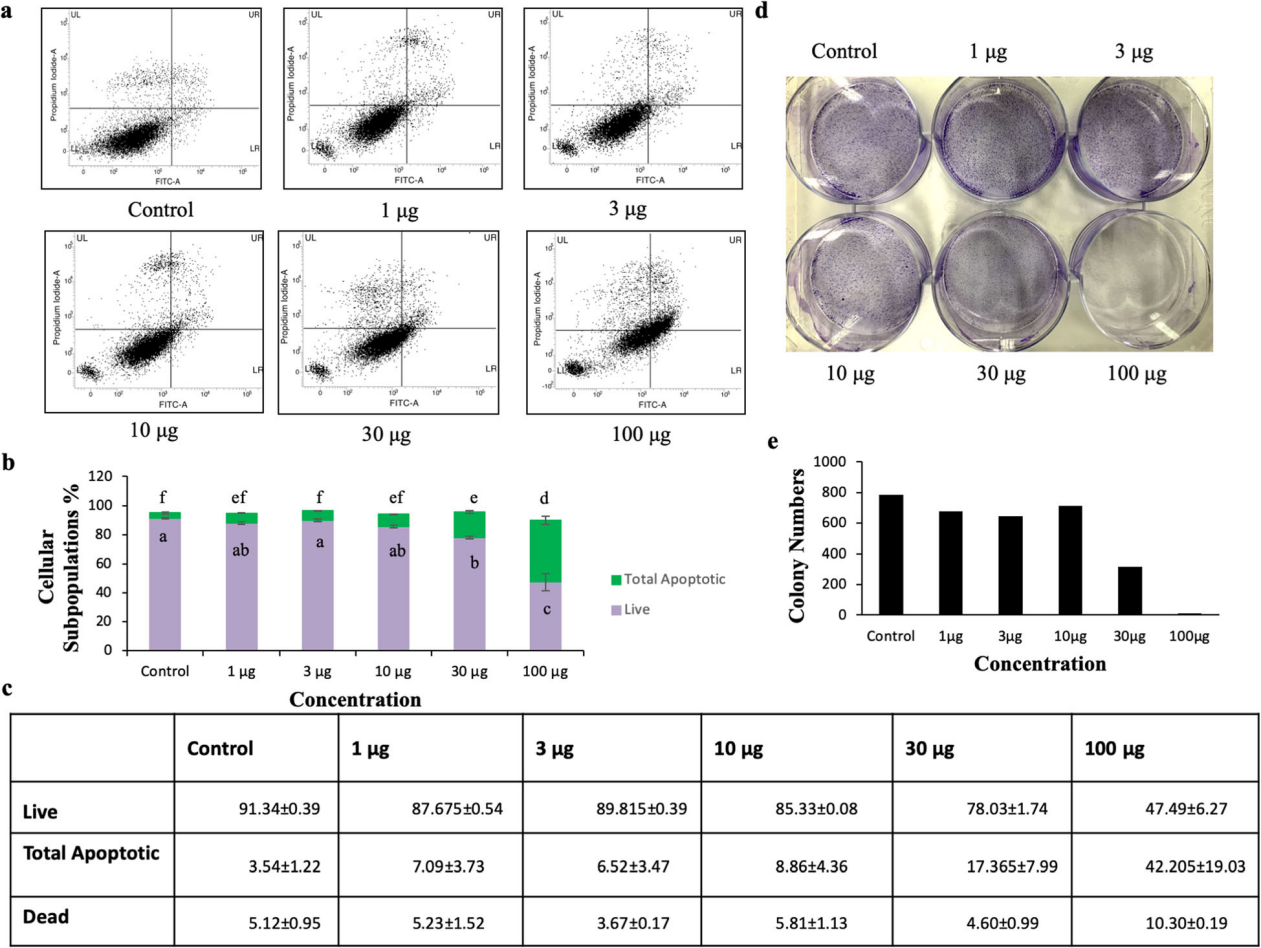
Evaluation of apoptosis induction in Hela cells after 48h of treatment with methanolic extract: **a** The histograms show the percentage of early and late apoptosis for one of the experiments. **b** The data are presented as the mean ± the SD (n=3), ^a~c^ and ^e~f^ values with different letters were significantly different at *p* < 0.05, as analyzed by one-way ANOVA followed by Turkeys post hoc test of significance. **c** The table shows the mean standard deviation (SD) percentage of live, early and late apoptotic cells from three independent experiments. **d** Colony formation was evaluated by clonogenic assay where cells were treated with indicated concentrations of the extract and (**e**) the colonies were quantified using ImageJ

The significant inhibition of Hela cells proliferation by ITM led us to check the effect on cell apoptosis. We found that the dose-dependent treatment of ITM caused the change in cellular morphology of cells (Fig. 4d). Quantitative estimation of apoptotic cells was carried out using FACS and the result of FACS analysis with double-positive annexin V-FITC/PI showed 42.2% (early apoptotic+late apoptotic population) in 100 μg/ml treated sample as compared to 3.5 % (early apoptotic+late apoptotic population) in vehicle control (Fig. 6a-c). We have shown dose-dependent effects of ITM on apoptosis (early and late) as well as standard deviation was calculated respectively.

To evaluate anti-proliferation activity in long-term cell culture and the ability of the cells to form colonies in the presence of ITM in Hela cells, colony formation assay was performed in a dose-dependent manner in the different time interval for 10 days. The outcome of the experiment was under the results obtained in the MTT assay and thus, a reduced clonogenic growth was observed in a dose-dependent manner with ITM, as shown in Fig. 6d. Densitometry analysis of no of colonies in each well with different doses of ITM was calculated by using Image J software as shown in Fig. 6e.

## Discussion

Phenolic compounds represent the largest category of natural products and are most widely distributed in the fungi. These are aromatic hydroxylated compounds with one or more hydroxyl groups and one or more aromatic rings [33]. They include phenolic acids and flavonoids amongst many and are widely reported to exhibit antioxidant activity in biological systems, acting as free radical inhibitors, metal inactivators, peroxide decomposers, or oxygen scavengers [34, 35]. According to previous reports, phenolics constitutes a major component of the fungal ingredients which is also a prominent antioxidant component reported in the members of mushrooms [36]. Thus, the total phenolics may be mainly responsible for antioxidant activity. Likewise, the extract also possesses flavonoids, which are capable of exerting anti-diabetic, anti-inflammatory, hepatoprotective, and cardioprotective properties [37, 38]. However, studies on flavonoid contents on mushrooms are scanty [39, 40]. The flavonoid content in the mycelium extracts during the present study reinforced its importance as potent antioxidant components. Furthermore, flavonoids are reported to possess strong antioxidant properties which could inhibit lipid peroxidation, scavenge free radicals, and chelate ferrous irons [41]. Therefore, owing to increasing demands for natural bioactive compounds in the pharmaceutical and food industries, it is imperative to use the mycelia of this fungus as a source of natural antioxidants. In *Macrocybe lobayensis*, an wild edible mushroom from the same locality, similar flavonoids content (10.95±0.89 μg QtE/mg) was observed, while the total phenol content in the same was way less (12.58 ±0.89 μg GAE/mg) like any other mushroom [42].

DPPH is a stable substrate used for the evaluation of the antioxidative activity. It is a free radical which accepts an electron or hydrogen radical to become a stable, diamagnetic molecule and produces a purple solution in methanol. It becomes pale in coloration when it reacts with antioxidant molecules and the DPPH radical which results in the scavenging of the radical by hydrogen donation [43]. The methanolic extract in the present study was able to reduce the purple solution to pale yellow colored diphenylpicrylhydrazine. The results align with the one determined with *Isaria sinclairii* where the pattern of result exhibited higher DPPH activity with increasing concentration [44]. In the DPPH assay, the use of solvent methanol might be responsible for better radical scavenging effects. Methanol has been widely reported for extracting hydrogen donating components from fungal samples, which can transfer a hydrogen atom to DPPH [45, 46]. The scavenging effects may, therefore, be partly attributed to proper solvent selection.

The ABTS assay effectively determines the antioxidant activity of hydrogen-donating and chain-breaking antioxidants [47]. The fungal resource is considered one of the indispensable sources of antioxidant, as it provides advantages over other plant materials as biomass in the form of mycelium, may be rapidly produced in the liquid culture and the composition of the culture medium can be manipulated to produce optimal quantities of bioactive products [47]. The findings from this study suggest that methanol extracts may possess certain metabolites that are useful as free radical inhibitors or scavengers in the cellular system. In the present study, the mycelium extract efficiently scavenged the ABTS radical in a dose-dependent manner indicating its possible usage as a scavenger of peroxyl radicals. This, results have correlated with an earlier study of antioxidant effects generated by two entomopathogenic fungi showing ABTS activity scavenging activity of 5.43 ±0.13 mg/ml and 6.29 ± 0.13 respectively [48].

Ferrous ions (Fe^2+^) chelation may render antioxidative effects by retarding metal-catalysed oxidation. Studies suggest that effective Fe^2+^ ion chelators might prevent oxidative damage by binding Fe^2+^ ions. Ferrozine form complexes with Fe^2+^ in the presence of chelating antioxidants. However, the complex formation is disrupted because of the interference of chelating agents which causes the disappearance of color. An earlier study on *Isaria farinosa* has proved the efficacy of low molecular weight exo-polysaccharide and intra-polysaccharides to chelate metal ions, where the chelating activity of *Isaria farinosa* was found to be 90.3% and 93.4% respectively at 12.8mg/ml [21]. Fe^2+^chelating effect to the tune of about 58% was shown by 700μg/ml extract of *Gomphus floccosus*, another edible wild mushroom from Eastern Himalaya [49]. The present findings are also in line with the experimental results found by Sharma [48] using exopolysachhrides and intrapolysaccharides isolated from *Isaria tenuipes*. Our works on *Isaria amoenerosea* have shown approximately 74% inhibition of Fe^2^ +[50] and therefore, the present work may be considered in sync.

Hydroxyl radical is considered to be the prime cause of lipid peroxidation and it may cross the membrane barrier, thereby readily reacting with macromolecules, such as carbohydrates, proteins, lipids, and DNA. It can also cause severe damage to these macromolecules that might result in cell death [51]. Thus, removing the excess of hydroxyl radicals from the living system is indispensable for the protection of vital functional molecules. The hydroxyl scavenging activity of the mycelial methanolic extract in the present study showed a moderate response that increased with an increase in the concentration of the extract. For a sample from the same genus (*Isaria farinosa*) reported elsewhere, the scavenging activity was around 46.48% when 3.2mg for water-soluble polysaccharide isolated from the species was used [21]. In the present study, a similar magnitude of the effect of about 50% inhibition of hydroxyl radical was obtained at 10mg/ml ITM extract. This result is consistent with the recent report on *Isaria amoenerosea* [50]. Thus, *Isaria tenuipes* may be considered a good scavenger of hydroxyl radicals also helping in preventing the damage of important macromolecules.

Vibrational spectrometry has been a widely used method for obtaining information on the chemical nature of potential bioactive substances. It helps to detect the vibrational frequencies and intensities of individual functional groups of the components in the natural mixture with high sensitivity and time resolution. It provides precisely very useful qualitative and quantitative information about the biochemical nature of antioxidants [51]. The mid-infrared region (from 4000 to 400 cm^-1^) was used in the present study to determine analytical information about chemical constituents present in methanolic extract. The selected wavelength provides meaningful information about the functional groups of molecules and has been widely used for the quantification of food components [52]. FTIR has been widely used to obtain vibrational spectra data of mycelium and fruit body of many medicinal mushrooms [53]. The FTIR spectra are useful for characterizing the functional groups of the molecules in the biological samples. The extract had characteristic IR bands that comprehensively indicate the presence of carbohydrates mainly polysaccharides, protein, and aromatic compounds. The peak recorded at 3292 cm^−1^ is the result of stretching vibrations of N-H and OH tentatively in the polysaccharides, triterpenes, and sterols [54]. The absorption around 2931 cm^−1^ (aliphatic C-H stretching, asymmetric) represent the hydrocarbon chain in the sample extract. The vibration at 1622 cm^−1^indicate the presence of proteins with amide I band which may be due to its covalent bonding with carbohydrates [55]. The absorption at 931cm^−1^is also characteristics of carbohydrates occurring in the mycelium extract. The present study preliminarily confirmed the presence of varied phytoconstituents with characteristic IR band in the mycelium extract. The chemical profile obtained in this study is comparable with data obtained by different workers from the different members of medicinal mushrooms [54, 55].

MTT assay was used to investigate the cell viability of HeLa, HepG2, and PC3 cells. ITM exposure resulted in significant dose-dependent inhibition of the growth of HeLa cells particularly. In the present study, it was recorded that percent inhibition was distinct only at relatively high extract concentration in the case of HepG2 and PC3 cells (>100μg/ml). Similar findings exhibiting a dose-dependent inhibition of cancer cells have also been reported for the allied genera [56]. The methanolic extract of entomopathogenic fungi of closely related genera *Paecilomyces* had shown significant cytotoxicity against human cancer lines mainly the HepG2 cell line [57]. Marine yeasts screened on human breast carcinoma cells (HepG2) were found to be cytotoxic with IC_50_ values of >31.25 μg/ml for all yeasts sample tested [58]. Similarly, fungal endophytes have also been reported to be cytotoxic against PC3 cell lines with an IC_50_ value of >50μg/ml [59]. In this context, the cytotoxic activity of the mycelial extracts established a fact that the entomopathogenic fungi are a sustainable source of natural anticancer compounds with potent activities. Further, the lead compounds present therein could also play a significant role in disease prevention and treatment. However, the antiproliferative effects may differ markedly based on different cancer cell types [60]. In brief, it is worth noting that the HeLa cells exhibited noticeable inhibition results at a lower concentration of extract tested. The inhibition of HeLa cells was found to be higher as compared to that of *Isaria amoenerosea* which showed about 53% inhibition [50].

**Fig. 7:**
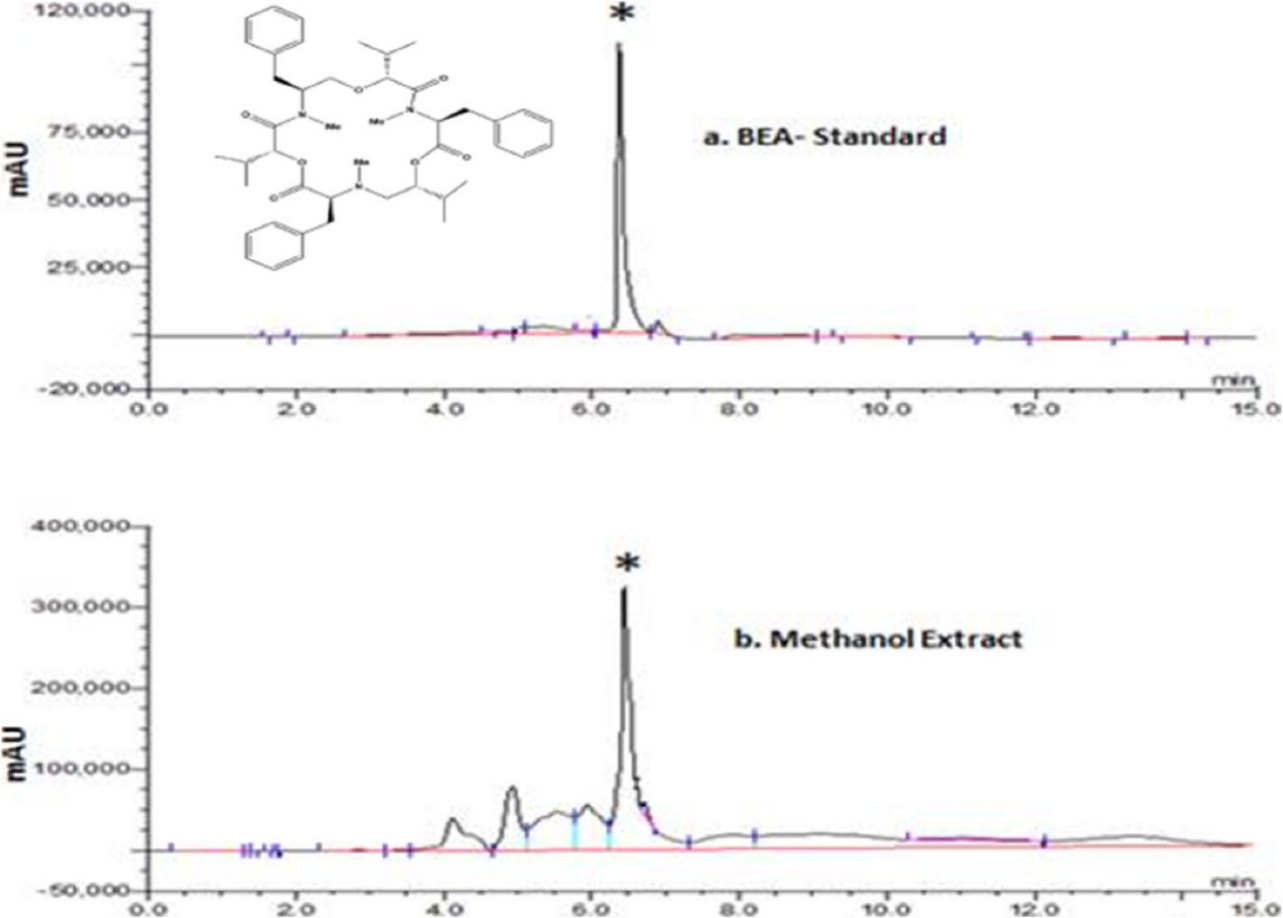
**a** HPLC profile representing signal generated at 210nm by injecting 20 μl of 20μg/ml beauvericin (BEA) standard; **b** methanol extract from mycelia

The results of the propidium iodide (PI) staining provided clear evidence for the apoptotic induction of cell death by the bioactive substances in the crude methanolic extract. Most possibly, cyclic peptides, mainly beauvericin isolated from fungal sources exert an anticancer effect [61]. Some studies on mangrove endophytic fungus have attributed the anticancer activities of beauvericin on several cancer cell lines including a human epidermoid carcinoma cell lines KB and KBv200 with an IC_50_ value of 5.76±0.55 and 5.34±0.09 μM, respectively [62]. Based on this experimentation, we also examined whether beauvericin, the active metabolite found in *Isaria spp.* was responsible for apoptosis. The presence of beauvericin was identified by a comparison of the retention time of methanolic extract with those of pure standard using HPLC. The HPLC chromatogram of the methanolic extract showed a major characteristic peak area at 6min which was in consonance to the peak of the internal standard used (Fig. 7). Earlier studies suggested that beauvericin had remarkable activity against diverse cancer cell lines with the potential for use as medicine [61]. The most important species of the genus *Isaria* which are reported to be a beauvericin producer includes *Isaria fumosorosea*, *Isaria japonica, Isaria cicadae, and Isaria tenuipes* [63]. Moreover, sterols isolated from *Cordyceps Sinensis* mainly sitosterol and ergosterol were found to possess apoptosis-inducing effects promyelocytic leukemia HL-60 cells [64]. *Cordyceps* species have long been exploited in alternative and complementary medicine and dietary therapy for cancer patients [65]. The present findings are in agreement with the recent studies on conidia of *Isaria cicadae* possessing beauvericin as one of the active ingredients inducing the caspase-mediated mitochondrial apoptosis pathway in gynecological carcinoma cells [66]. Further, the presence of cordycepin which is a strong anti-cancer agent has already been reported from *Isaria japonica* [67]. Cordycepin inhibits cell proliferation by influencing the signaling caspase pathway [68]. The presence of this metabolite may not be ruled out in this species too. However, further studies may throw light in this area. Therefore, in the present work, we believe that the presence of secondary metabolite chiefly the beauvericin along with other myco-constituents as revealed through FTIR spectra might be responsible to a certain extent for bringing about cytotoxicity to HeLa cells in a dose-dependent manner.

Cells undergoing the process of apoptosis after proper stimulation by the trigger molecules experience many typical biochemical and morphological changes in a cascade [69]. Morphological investigation of the methanolic extract at 100 μg/ml concentration revealed cell death through the mechanism of apoptosis (Fig. 4d). Apoptosis involves the activation of characteristics pathway that leads to programmed cell death or leads to the suicide of the cell by a characteristics process in which the cell exhibit certain cellular morphology under the microscope [70]. As compared to the untreated cells, the treated cells became compact which had shrunken irregular margin and a high degree of membrane blebs indicating the formation of apoptotic bodies. From such evaluation, it could be presumed that ITM may be responsible for bringing about apoptosis under in vitro conditions. Recent studies suggest that *Isaria tenuipes* is rich in various secondary metabolites making it an important fungal species with potential pharmacological functions [71]. Further research is hence needed to unravel the mechanistic pathways involved in cytotoxicity as this study was only conducted on crude methanol extract for initial screening purposes.

## Conclusion

The current investigation with the methanolic extracts of the mycelium of *Isaria tenuipes* presents the first report on this species from this region. The methanolic extract of the fungus exhibited quite a significant quantity of phenols and flavonoids which are related to its antioxidant activities and can inhibit the proliferation of cancer cells. Besides, the active principle, beauvericin may have an additive effect, which was also indicated by the flow cytometric studies on cytotoxicity of the extract against a cancer cell line. Further, it can be concluded that the crude methanolic extract initiates cell death via apoptosis. However, it may be noted that the cytotoxic activity of fungal extracts may vary with the varying cell types and the biological activities were directly proportional to the extract concentrations. It can be concluded that the crude methanolic extract exhibited potent antioxidant and antiproliferative activities suggesting natural antioxidative and antiproliferative agents, which need further study to develop cancer therapeutic adjuvant.

## Data Availability

The datasets used and/or analyzed during the current study are available from the first author on reasonable request.
